# Introducing Long-Acting Contraceptive Removal Indicators in a Pilot Study in Mozambique: Dynamics of Discontinuation and Implications for Quality of Care

**DOI:** 10.9745/GHSP-D-21-00252

**Published:** 2022-02-28

**Authors:** Ana Jacinto, Adalgisa Viola Ronda, Connie Lee, Fariyal F. Fikree, Eric Ramirez-Ferrero

**Affiliations:** a Pathfinder International, Maputo, Mozambique.; b Pathfinder International, Washington, DC, USA.; c Consultant, Washington, DC, USA.; d MOMENTUM Integrated Health Resilience Project, Corus International, Washington, DC, USA; formerly of Pathfinder International, Washington, DC, USA.

## Abstract

**Introduction:**

The recent increase in the use of long-acting reversible contraceptives (LARCs) will result in an equal increase in the need for removals. Few countries monitor the number of LARC removals and even fewer track reasons for removal, discontinuation, and method switching. We conducted a mixed-method study to pilot the introduction of 5 LARC removal indicators in Mozambique: (1) reason for seeking removal, (2) duration of use, (3) removal outcome, (4) reason for referral, and (5) family planning (FP) outcome. We assessed providers’ perceptions about the feasibility and utility of tracking these indicators and reviewed the findings to ascertain the quality-of-care concerns.

**Methods:**

We used a purposive, multistage sampling technique to select 19 intervention facilities in Nampula and Sofala provinces. Over 6 months, we abstracted data on the 5 indicators from an FP register addendum to determine when and why clients seek LARC removals and identify service quality issues that need to be addressed in the FP program. We used a supportive supervision checklist to assess the time taken to record data on the indicators and perceived benefits and challenges encountered by providers during record keeping.

**Results:**

Of the 795 clients who sought a LARC removal, 112 women (14.1%) opted not to have the removal after counseling. The most frequently reported reasons for seeking a removal were: the method was on-schedule for removal or past its expiration date (29.5%), complaints of side effects (25.8%), and desire to be pregnant (22.9%). Health care providers reported no major challenges in recording information in the addendum. Reasons for and timing of removal pointed to the need for strengthening FP counseling.

**Conclusion:**

Collecting, synthesizing, and interpreting data from the LARC removal indicators was feasible and provided valuable insights to improve the quality of care to enhance clients’ reproductive health care and contraceptive choices.

## Abstract (precis)

Tracking information about long-acting reversible contraceptive removals in the national health management information system is feasible and useful for improving the quality of family planning services in Mozambique.

## Teaser

**Key Findings:**

- Synthesis and interpretation of the long-acting reversible contraceptive (LARC) removals data underscored the need to strengthen family planning (FP) counseling, particularly on method side effects and use-effectiveness periods, in all client-provider interactions.
- Health care providers perceived “the addendum with LARC removal indicators” as easy to use and useful for understanding quality-of-care issues.

**Key Implications:**

- National stakeholders and program implementers should ensure routine and robust FP counseling at all client-provider interactions, including before inserting of LARCs and time of removal, to better enable clients to make informed method choices.
- Governments should consider including LARC removal indicators in their national health management information system to strengthen monitoring of the FP program and the delivery of high-quality FP care.

Resumo em português no final do artigo.

## INTRODUCTION

Since 2012, client uptake of long-acting reversible contraceptive (LARC) in sub-Saharan Africa (SSA) has steadily increased.^1^^,^^2^ Client uptake of LARCs has been higher in selected East and Southern African countries compared to those in West and Central Africa.^1^ Over a 10-year review period (2003–2017), prevalence of intrauterine devices (IUDs) has been low but on a modest upward trajectory, with gains ranging from 0.3 to 1.6 percentage points across 11 SSA countries.^2^ Increases in implant contraceptive prevalence rate (CPR) in SSA have been more substantial, with Kenya experiencing the most dramatic rise: from 1.7% in 2003 to 18.1% in 2016.^2^ The rapid gains in implant use have been evenly distributed across almost all sociodemographic categories in many SSA countries.^2^ Jacobstein argued that this rise in implant CPR was associated with^2^:

*… sizeable price reductions, much-increased commodity supply, greater government commitment to rights-based family planning, broader [World Health Organization] eligibility guidance, and wider adoption of high-impact service delivery practices …*

The increases in implant access and use must be accompanied by implant removal services that are available, accessible, and affordable, as all inserted implants must ultimately be removed by technically competent providers. Consequently, consistent and dependable access to insertion and removal services will be paramount in sustaining this success. However, Christofield and Lacoste^3^ noted that the rapid increase in client uptake of implants in recent years has not been matched with commensurate attention to implant removals.

Emerging data suggest a gap between the client uptake of implants and service delivery capacity for implant removals,^3^ raising issues of accessibility and quality of removal services, as well as an absence of data to drive quality improvement. One condition of service quality is the availability of health care providers who are competent in standard and difficult LARC removals. Difficult removals are rare occurrences involving implant rod(s) that are deeply palpable or not palpable at all, IUDs whose strings cannot be visualized, or devices that become difficult to remove during the procedure.^4^ For difficult removals, the implant rod(s) or IUD may need to be localized with ultrasound before removal and require skilled providers experienced in difficult removals.^4^ Although many providers are trained in removals, they may not use their skills often enough to maintain their competence and confidence in performing the procedure due to low patient volume, particularly for difficult removals, insufficient supplies and equipment at health facilities, or unclear systems and processes for managing difficult removals.

Although many providers are trained in implant removals, they may not use their skills often enough to maintain their competence and confidence in performing the procedure.

Another important service quality area is effective counseling of women seeking removals that addresses their specific concerns. Although LARC discontinuation rates are much lower than rates for short-acting contraceptive methods,^5^ women remain concerned about side effects of all contraceptive methods and express beliefs in myths and misperceptions about contraception.^5^ Quality counseling that addresses these concerns and routine follow-up care can largely curb these contributors to early discontinuation, particularly among women who opt for LARCs.^6^^–^^11^ Technically competent health care providers and the appropriate constellation of counseling, service delivery, and follow-up care for LARC insertions and removals, available in the same locality, are key to satisfied clients and future continued use. This is important because inadequate availability and accessibility of LARC removal services deny women the opportunity to make an informed and voluntary contraceptive choice, aligned with their reproductive intentions, to discontinue use, irrespective of age, parity, or marital status.

In addition to service delivery gaps, few countries routinely monitor LARC removals in their health management information system (HMIS), and even fewer track reasons for removal, discontinuation rates, and rates of method switching. This absence of data on removals hinders ministries and program managers’ ability to monitor and appropriately address quality-of-care concerns^5^^,^^12^ and has the potential to limit their accountability to provide the full range of service, including follow-up and removal, to meet client demand.^3^

Recognizing the need for more attention on removals, the Implant Access Program^13^ initiated the Implants Removals Task Force in 2015 to ensure access to quality implant removal services and identified data, research, and programming needs. The Task Force developed a list of 8 conditions that constitute quality implant removal services,^14^ 1 of which is that implant removal data are collected and monitored. Subsequently, the Task Force’s data subcommittee recommended that countries monitor 6 LARC removal indicators. These included: (1) reason for client visit, (2) reason for seeking removal, (3) duration of use, (4) removal outcome, (5) reason for referral (if implants not removed/referred for removal), and (6) family planning (FP) outcome. The expectation is that these indicators could help track LARC removal quality-of-care components within national FP HMIS, identify problems or weaknesses, and enable action to address them.

As a Task Force member and lead partner of the data subcommittee, the Evidence to Action (E2A) Project initiated a collaboration with the U.S. Agency for International Development (USAID)/Mozambique-supported Integrated Family Planning Project (IFPP) to assess the feasibility and utility of including LARC removal indicators in the national FP HMIS. This article describes the experience of implementing an addendum to the national FP register at study facilities containing 5 of the 6 Task Force-recommended indicators and illustrates its utility in ascertaining the quality-of-care concerns to guide programmatic adjustments. The indicator “reason for clinic visit” was not included in the addendum as it is already captured in the FP register. The method uptake portion of the “FP outcome” indicator also appears in the register but was duplicated in the addendum to facilitate data collection for the study. In addition, this article describes health care providers’ perspectives about the feasibility and usefulness of including these indicators for improving service delivery. For this article, the indicators track removals of the 2-rod levonorgestrel implant (Jadelle), the 1-rod etonogestrel implant (Implanon NXT), and copper-T IUD, the only LARCs available at all public-sector facilities in Mozambique.

## METHODS

### Design and Study Setting

Conducted from December 2018 to May 2019, this mixed-methods study used quantitative data extracted from the FP register addendum for 6 months as well as qualitative data collected from health care provider interviews during monthly supportive supervision visits regarding their perspectives on the feasibility and utility of the removal indicator suite. E2A obtained ethical approval for this study from the Bioethics Committee of the Ministry of Health (MOH) in Mozambique, Mozambique Institutional Review Board #00002657, registration number 88/CNBS/2018. In the United States, PATH’s Research Determination Committee determined that the study did not meet the definition of human subjects research as per U.S. federal regulations and thus did not require review by PATH’s Institutional Review Board.

Mozambique has a modern CPR of 25.3%; IUD and implant CPR are 0.8% and 1.7%, respectively (Table 1).^15^ Between 2013 and 2018, the total number of annual implant insertions rose from 32,327 to 324,072 as reported in the District Health Information Software 2 (DHIS2).^16^ This study was conducted in Nampula and Sofala provinces of Mozambique where USAID/Mozambique’s IFPP project, implemented by Pathfinder International and its consortium partners, aimed to increase the modern CPR in Mozambique by strengthening FP counseling and service provision at the 402 public-sector health facilities in the 2 provinces (Nampula=238; Sofala=164). All contraceptive methods, including LARCs, were free of charge at these facilities. The provinces were selected based on feasibility and convenience, enabling day-to-day project oversight.

**TABLE 1:** Modern CPR and Long-Acting Reversible Contraceptive CPR, Nampula and Sofala Provinces, Mozambique, 2015

	Modern CPR%	IUD CPR%	Implant CPR%
Mozambique	25.5	0.8	1.7
Nampula	21.8	0.3	1.0
Sofala	14.4	0.5	1.3

Abbreviations: CPR, contraceptive prevalence rate, IUD, intrauterine device.

### Sampling

The study used a purposive, multistage sampling technique to select the districts and health centers in each province. In the first stage, we reviewed national HMIS data and extracted the number of LARC removal clients for 15 months (January 2017–March 2018) from the registers of the 402 health facilities in Nampula and Sofala. We selected district hospitals reporting removals (Nampula=6; Sofala=5) and aligned each district hospital with its respective health centers (Nampula=182; Sofala=61) located within the district hospital’s catchment area. In the second stage, we selected health centers that reported 5 or more removals in 1 or more months over the same 15-month period, reducing the total number of health centers (Nampula=8; Sofala=9) and aligned districts (Nampula=5; Sofala=4) per province. In the final stage of sampling, we selected 2 health centers per district that reported the highest number of removals in any month. The total number of study sites selected was 17 (Nampula=8; Sofala=9). Provincial MOH stakeholders recommended including provincial hospitals, yielding a final sample of 19 facilities—10 urban facilities (2 provincial hospitals, 4 district hospitals, 4 urban health centers) and 9 rural health centers.

### Tool Description

We collected data from 3 sources that were introduced during a training of service providers in early November 2018: 2 FP register addendums (1 for standards removals, 1 for difficult removals) and a supportive supervision checklist. The addendum for standard removals (Supplement 1) included 5 LARC removal indicators (Box): reason for seeking removal, duration of use, removal outcome, reason for referral, and FP outcome. The addendum for difficult removals included these same indicators except for reason for seeking removal. Both addendums included client’s current age, parity, and marital status. All study facilities implemented the standards removal addendum, while only referral sites (4 district and 2 provincial hospitals) implemented the difficult removal addendum.

BOX **Description of Long-Acting Reversible Contraceptive (LARC) Removal Indicators**

**Reason for seeking removal** (only in standard removal addendum): Options included on-schedule/expired (method used for its full labeled duration or beyond), method change, desire to get pregnant, vaginal bleeding, vaginal discharge, arm pain, backache, headache, family opposition, infrequent sex, reduces sexual pleasure, interferes with body natural processes, method failure, intrauterine device (IUD) expulsion. Multiple response instructions were also included.

Health providers recorded the codes corresponding with the reasons in the order given by clients and were able to use these stated reasons to inform the counseling provided. For study purposes, we only extracted and reviewed the first reason women gave, and recoded them under 8 broad categories: (1) side effects (vaginal bleeding, headache, vaginal discharge, weight gain or loss); (2) other adverse events (arm discomfort, back pain); (3) family opposition (mother/husband/in-laws); (4) desire to be pregnant; (5) on schedule/expired (used for within 4 months of the full duration of labeled effectiveness, or beyond); (6) method failure (became pregnant while using the method); (7) switch method; (8) and other, including need for hysterectomy. While asking clients about reason for removal could be perceived as compromising their contraceptive autonomy, we reasoned that including the indicator may unveil valuable opportunities for program improvements to help ensure women are able to make voluntary and informed method choices.

**Duration of use**: Time interval in months from date of insertion to date of successful removal. If day or month was not recorded for date of insertion, we assigned “15” for day and “6” for month; if the year was not recorded, we assigned “missing.” We subsequently recategorized duration of use into 4 categories.

 1.  Within the first 3 months: Removals completed within 0–3 months from the time of method insertion. We included this category to serve as an important marker for clinical and programmatic follow-up, indicating a potential gap in quality of care at the time of insertion.

 2.  On schedule/expired:

  •  On schedule: Within the 4 months before each method’s labeled duration of effectiveness: (33–36 months for etonogestrel implants; 57–60 months for levonorgestrel implants, and 117–120 months for IUD).

  •  Expired: End of the labeled duration of use.

 3.  Before expiration: Removals completed from the fourth month after insertion up to 4 months before the method’s expiration date: (4–32 months for etonogestrel implants, 4–56 months for levonorgestrel implants, and 4–116 months for IUD).

 4.  After expiration: all removals completed after the labeled duration of effectiveness for each method.

**Removal outcome**: Options included: not removed following counseling, not removed and referred, attempted but not removed and referred, and removed.

  •  Attempted but not removed: Removals that were not completed due to difficulties encountered during the client visit. These incomplete removals required referral to a higher-level facility.

  •  Removed: Included a series of options to describe the removal process (no difficulty, with difficulty, with significant difficulty) to capture the range of potential outcomes during the removal process. With significant difficulty indicates that surgery was necessary to remove the method.

**Reason for referral:** Options included: trained provider unavailable, equipment/consumables not available, implant unpalpable, IUD string not visible, removal attempted but not completed, complicated removal (additional equipment/expertise required), and referral requested by the client. Multiple response instructions were also included. When multiple responses were recorded in the addendum (in the order given by the client), we extracted and analyzed only the first response for this study.

**FP outcome:** Addendum included 2 columns to indicate FP counseling provided and FP uptake post-removal. Supplement 3 depicts the decision-making process for the FP outcome indicator recorded in these 2 columns.

  •  **FP counseling:** not counseled, counseled only, counseled and chose a method, counseled and referred for another method, counseled and refused another method

  •  **FP method uptake:** LARCs, short-acting methods, and tubal ligation

The supportive supervision checklist (Supplement 2) was a structured tool with 9 open-ended questions for providers that explored the perceived benefits and challenges encountered in completing the standard removal addendum and time taken in recording these indicators.

### Study Implementation

Four study team members from IFPP’s offices in Nampula and Sofala participated in a 2-day training workshop conducted by E2A and IFPP. The FP addendum and supportive supervision checklist were pretested on the second day to determine the clarity, flow, and cultural appropriateness of the questions. Based on pretest observations, we revised and reworded the tools as needed, then translated the tools into Portuguese. In early November 2018, the team conducted a 1-day training with 47 health care providers who work in the FP consultation rooms from the 19 study facilities and 12 technical staff from the provincial health directorates on how to record data in the FP addendums. This was immediately followed by a 2-week field trial run to resolve any issues with data recording and improve data quality. Data collection commenced thereafter, aligned with HMIS monthly reporting, starting November 21, 2018, to May 20, 2019 (herein referred to as December 2018–May 2019).

After the close of an HMIS reporting month, a study team member visited each facility to review the previous month’s FP register and addendum with the trained health care provider, address data entry or data quality issues, and scan the addendum pages. Relevant data (age, parity, marital status, and LARC removal indicators) were extracted from the scanned pages and transferred to Microsoft Excel to ensure standardized data extraction and data entry across study sites. During the supervision visit to the facility, a study team member used the supportive supervision checklist to interview the trained health care provider who was on duty. These interviews aimed to gauge the feasibility and usefulness of introducing the LARC removal indicators from the perspective of providers.

Between December 2018 and May 2019, we documented a total of 795 LARC removal clients at 18 facilities—614 implant, 68 IUD, and 113 unknown method users. (The addendum did not capture the methods of clients who did not have a removal during their visit.) Accessibility to 1 health facility in Sofala was markedly curtailed due to heavy rains that damaged the main roads to the facility. As a result, this facility was dropped from the analysis. In the remaining 18 study sites, data collection continued but was delayed in 6 Sofala study sites for approximately 3 weeks due to Hurricane Idai. Regarding qualitative data collection challenges, although monthly supervision visits were planned for each facility, only 64 supervisory visits (Nampula=33; Sofala=31) across all study sites were conducted over the study period due to logistics and other related accessibility challenges.

### Data Analysis

To review the data from the FP addendum, we generated frequency tables using SPSS Version 22 with the aggregated 6-month distribution of results for the following indicators: reason for client visit, reason for seeking removal, duration of use, removal outcome, reason for referral, and FP outcome, disaggregated by province and current method of use. We also reviewed and thematically analyzed information from the supportive supervision checklist to assess providers’ perspectives on the benefits of collecting LARC removal data, including their usefulness to improve quality of care, as well as feasibility, including ease of recording the removal indicator suite, time taken to record, perceived burden, and challenges encountered during record keeping.

## RESULTS

### Characteristics of Clients Seeking LARC Removal

Of the 71,027 clients who accessed the 18 health facilities in Nampula and Sofala for FP services during the study period, 42,198 clients chose a contraceptive method and 8,276 chose a LARC method. The number of clients who sought a LARC removal and were thus included in the study sample was 795 (1.1% of all clients and 9.6% of LARC adopters). Table 2 describes characteristics of these LARC removal clients by province, according to 3 demographic variables: age, marital status, and parity. Many of the clients were young (42.6% under 25 years), unmarried/not in union (43.8%), and either multiparous (37.5%) or low parity (51.3%). While age and parity characteristics were similar across both provinces, there were statistically significant provincial differences in terms of marital status. Only 38% of clients were married or living in union in Nampula, unlike Sofala where nearly 65% were married or living in union (P<.00).

**TABLE 2.** Demographic Characteristics of LARC Removal Clients,^a^ Nampula and Sofala Provinces, Mozambique, December 2018–May 2019

	**Nampula (n=336) No. (%)**	**Sofala (n=459) No. (%)**	**Total (N=795) No. (%)**
Age, years (N=791)			
≤19	46 (13.7)	52 (11.4)	98 (12.4)
20–24	93 (27.8)	147 (32.2)	240 (30.2)
25–29	94 (28.1)	121 (26.5)	215 (27.2)
30–34	51 (15.2)	71 (15.6)	122 (15.4)
≥35	51 (15.2)	65 (14.3)	116 (14.7)
Marital status (N=792)			
Married	88 (26.3)	260 (56.9)	348 (43.9)
Living in union	40 (11.9)	36 (7.9)	76 (9.6)
Divorced/separated/widowed	9 (2.7)	12 (2.6)	21 (2.6)
Never married nor lived in union	198 (59.1)	149 (32.6)	347 (43.8)
Parity (N=794)			
Nulliparous	58 (17.8)	31 (6.8)	89 (11.2)
1–2	143 (42.6)	264 (57.6)	407 (51.3)
3+	135 (40.2)	163 (35.6)	298 (37.5)
Method			
Two-rod levonorgestrel implant	251 (74.7)	342 (74.5)	593 (74.6)
One-rod etonogestrel implant	10 (3.0)	11 (2.4)	21 (2.6)
Intrauterine device (copper)	38 (11.3)	30 (6.5)	68 (8.6)
Method unknown^b^	37 (11.0)	76 (16.6)	113 (14.2)

^a^ Missing information: Nampula: age=1, marital status=1; Sofala: age=3, marital status=2, parity=1.

^b^ Method of use at the time of the visit was not captured in the standard removal addendum for clients whose method was not removed.

In addition to these characteristics, we noted the type of health facility that clients visited for LARC removals (Table 3). The majority (86%) of clients sought services at the facilities located in urban or peri-urban areas (health centers, district hospitals, or provincial hospitals).

**TABLE 3.** Type of Facilities Visited by Clients Seeking Long-Acting Reversible Contraceptive Method Removal and Reported Reason for Removal, Nampula and Sofala Provinces, Mozambique, December 2018–May 2019

	**Nampula (n=336)** **No. (%)**	**Sofala (n=459) No. (%)**	**Total (N=795) No. (%)**
Facility category			
Provincial hospital	32 (9.5)	29 (6.3)	61 (7.7)
District hospital	35 (10.4)	114 (24.8)	149 (18.7)
Urban health center	227 (67.6)	247 (53.8)	474 (59.6)
Rural health center	42 (12.5)	69 (15.0)	111 (14.2)
Reason for removal^a^^,b^			
On schedule/expired	92 (28.2)	136 (30.5)	228 (29.5)
Side effects^c^	96 (29.4)	103 (23.1)	199 (25.8)
Desire to be pregnant	58 (17.8)	119 (26.7)	177 (22.9)
Switch method	29 (8.9)	32 (7.2)	61 (7.9)
Other adverse events^d^	32 (9.8)	11 (2.5)	43 (5.6)
Family opposition^e^	6 (1.8)	19 (4.3)	25 (3.2)
Method failure	5 (1.5)	8 (1.8)	13 (1.7)
Others	8 (2.5)	18 (4.0)	26 (3.4)

^a^ N=772. When multiple responses were given, only the first reason was recorded.

^b^ Missing information: Nampula: Reason for Removal=10; Sofala: Reason for Removal=13.

^c^ Vaginal bleeding, vaginal discharge, headache, or weight gain or loss.

^d^ Arm discomfort/pain or back pain.

^e^ Husband/in-law opposition or mother opposition.

### Reason for Seeking Removal

Also shown in Table 3, the 3 most frequently reported reasons for seeking LARC removal were on schedule/expired (29.5%), side effects (25.8%), and desire to be pregnant (22.9%). Close to 8% of clients sought removals to switch methods. Vaginal bleeding, a common contraceptive method side effect, was the most frequently reported side effect (n=166).

### Duration of Use

Complete or assigned date of insertion was available for 661 (83%) clients seeking LARC removal, enabling the calculation of duration of use (Table 4). Of these, the majority (87.3%, n=577) sought removals before their method’s corresponding expiration dates, contrasting with the 29.5% of clients claiming “on schedule/expired” as their reason for removal. Few clients (5.6%, n=37) had true “on schedule” removals, all of whom were levonorgestrel implant users. There were substantial numbers of removals in the first 3 months of use, with proportionally more reported for the etonogestrel implant (42.1%, n=8) and IUD (24.2%, n=16) than for the levonorgestrel implant (9.9%, n=57). Only 7% (n=47) of the clients came in for removals after their method’s use-effectiveness period, mostly among levonorgestrel implant users.

**TABLE 4.** Duration of Use by Long-Acting Reversible Contraceptive Method, Nampula and Sofala Provinces, Mozambique, December 2018–May 2019

**Duration of Use**	**Nampula, No. (%)**	**Sofala, No. (%)**	**Total, No. (%)**
One-rod etonogestrel (n=19)			
Within first 3 months	6 (66.7)	2 (20.0)	8 (42.1)
Before expiration	3 (33.3)	7 (70.0)	10 (52.6)
On schedule/expired (33–36 months)	0 (0)	0 (0)	0 (0)
After expiration	0 (0)	1 (10.0)	1 (5.3)
Two-rod levonorgestrel implant (n=576)			
Within first 3 months	29 (11.7)	28 (8.5)	57 (9.9)
Before expiration	179 (72.5)	259 (78.7)	438 (76.0)
On schedule/expired (57–60 months)	17 (6.9)	20 (6.1)	37 (6.4)
After expiration	22 (8.9)	22 (6.7)	44 (7.6)
Intrauterine device (copper) (n=66)			
Within first 3 months	14 (37.8)	2 (6.9)	16 (24.2)
Before expiration	21 (56.8)	27 (93.1)	48 (72.7)
On schedule/expired (117–120 months)	0 (0)	0 (0)	0 (0)
After expiration	2 (5.4)	0 (0)	2 (3.0)
All methods (n=661)			
Within first 3 months	49 (16.7)	32 (8.7)	81 (12.3)
Before expiration	203 (69.3)	293 (79.6)	496 (75.0)
On schedule/expired	17 (5.8)	20 (5.4)	37 (5.6)
After expiration	24 (8.2)	23 (6.3)	47 (7.1)

### Removal Outcome

Table 5 describes the distribution of clients based on removal outcome, by their current method of use. Of the 795 women who sought removal services, 112 women (14.1%) changed their minds and opted not to go through with the removal after an initial targeted counseling session. The primary reasons for removal among these 112 women were side effects (45%) and on schedule/expired (16%). The majority (88%) of the 112 clients reported at the time of visit, they had used the method for 3 months or less. This result reinforces the critical role of counseling at initial client contact as well as during follow-up care. More than four-fifths of all clients seeking removal services (83.5%, n=664) successfully had LARCs removed with no difficulty, while much fewer had LARCs removed with difficulty (1.3%, n=10). Among the 674 successful removals (removed with no difficulty and removed with difficulty), most were implants (levonorgestrel: n=588; etonogestrel: n=21); there were 66 IUDs removed. No cases of significantly difficult removals were recorded in the standard removal addendum. However, 5 cases were marked as attempted but not removed plus an additional 4 cases were marked for referral.

**TABLE 5.** Removal Outcome by Long-Acting Reversible Contraceptive Method, Nampula and Sofala Provinces, Mozambique, December 2018–May 2019

	**Levonorgestrel Implant (n=593)** **No. (%)**	**Etonogestrel Implant (n=21)** **No. (%)**	**Copper Intrauterine Device (n=67)** **No. (%)**	**Unknown Method**^a^ **(n=114)** **No. (%)**	**Total** **(N=795)** **No. (%)**
Not removed following counseling	--	--	--	112 (98.2)	112 (14.1)
Removed: no difficulty	579 (97.6)	21 (100)	64 (95.5)	--	664 (83.5)
Removed: with difficulty	9 (1.5)	0 (0)	1 (1.5)	--	10 (1.3)
Removed: significant difficulty	0 (0)	0 (0)	0 (0)	--	0 (0)
Attempted but not removed: referred	4 (0.7)	0 (0)	1 (1.5)	--	5^b^ (0.6)
Not removed: referred	1 (0.2)	0 (0)	1 (1.5)	2^c^ (1.8)	4 (0.5)

^a^ Method of use at the time of the visit was not captured in the standard removal addendum for clients whose method was not removed.

^b^ Two clients were referred for removal; data missing for three clients about referral or rescheduled appointment.

^c^ Missing data for two clients who were referred for difficult removals.

Of the clients who opted not to remove their LARC, 88% reported they had used the method for 3 months or less.

### Reason for Referral

As noted in Table 5, only 4 referrals were registered in the standard removal addendum. Reasons for these referrals included IUD string not visible, equipment/consumables unavailable, and implant not palpable/removal too complicated. However, there were also 5 cases registered as attempted but not removed, with 2 of these subsequently marked as referred. Although a total of 6 clients were marked as referred, the difficult removal addendum, implemented at district and provincial hospitals to track referrals and their outcomes, did not record any difficult removals. It was not possible to track the medical record number and/or referral form for these 6 clients at the referral facilities to ensure successful removals, despite all efforts by the field team.

### FP Outcome

Of the 674 clients who had their LARC method successfully removed, information on FP services offered after removal was available for 673 clients (Table 6). Just over one-third of these clients were counseled and chose a method, thus categorized as FP users. The majority of these FP users (80.3%, n=187) switched to short-acting reversible contraceptives, while another 16.7% (n=39) decided to continue using a LARC method and 3% (n=7) chose tubal ligation. Counseling services after removal were not provided to 188 clients (27.9%); although 127 (67%) of these women were not counseled because they desired a pregnancy (noted as reason for removal), it is not clear from this study why the remaining women were not counseled, signaling a potential service quality concern. Approximately 8% (n=52) were only provided with FP counseling but not offered a contraceptive during the visit nor provided with a referral.

**TABLE 6.** Family Planning Outcomes of Clients Who Had Long-Acting Reversible Contraceptive Method Successfully Removed, Nampula and Sofala Provinces, Mozambique, December 2018–May 2019

	**Nampula (n=302)**	**Sofala (n=371)**	**Total (N=673)**
	**No. (%)**	**No. (%)**	**No. (%)**
Family planning counseling provision			
Not counseled	46 (15.2)	142 (38.3)	188 (27.9)
Counseled only	16 (5.3)	36 (9.7)	52 (7.7)
Counseled and chose a method	141 (46.7)	92 (24.8)	233 (34.6)
Counseled and referred for a method	2 (0.7)	1 (0.3)	3 (0.4)
Counseled and refused a method	97 (32.1)	100 (27.0)	197 (29.3)
Method uptake by client			
Long-acting reversible contraceptive (continuers)	18 (12.8)	21 (22.8)	39 (16.7)
Short-acting reversible contraceptive (switchers)	116 (82.3)	71 (77.2)	187 (80.3)
Tubal ligation (switchers)	7 (5.0)	0 (0)	7 (3.0)

As shown in Table 7, among the 39 removal clients who opted to continue using a LARC method, the majority (64%, n=25) selected levonorgestrel implant. Six LARCs continuers switched from IUD to implants, 10 switched from implant to IUD, while 23 implant users decided to continue with an implant.

**TABLE 7.** Clients Who Had LARC Method Removed and Selected a LARC, Nampula and Sofala Provinces, Mozambique, December 2018–May 2019

	Method Removed			
	Levonorgestrel Implant (n=33) No. (%)	Etonogestrel Implant (n=0) No. (%)	Copper IUD (n=6) No. (%)	Total (N=39) No. (%)
Selected levonorgestrel implant	21 (63.6)	0 (0)	4 (66.7)	25 (64.1)
Selected etonogestrel implant	2 (6.1)	0 (0)	2 (33.3)	4 (10.3)
Selected copper IUD	10 (30.3)	0 (0)	0 (0)	10 (25.6)

Abbreviations: IUD, intrauterine device, LARC, long-acting reversible contraceptive.

We also examined the relationship between vaginal bleeding and continued FP use (Table 8). Of the 166 clients reporting vaginal bleeding as the primary reason for wanting their LARC removed, 26.5% decided not to remove their LARC after counseling, 1.8% continued with a different LARC method, 36.8% switched to a short-acting hormonal method after removal (oral pills=31; injectables=27), and 34.9% chose to not use a contraceptive method after LARC removal.

**TABLE 8.** FP Status of Clients Who Sought LARC Method Removal for Vaginal Bleeding, Nampula and Sofala Provinces, Mozambique, December 2018–May 2019

	**Nampula (n=80)** **No. (%)**	**Sofala (n=86)** **No. (%)**	**Total (N=166)** **No. (%)**
Non-user	31 (38.7)	27 (31.4)	58 (34.9)
LARCs (not removed)	14 (17.5)	30 (34.9)	44 (26.5)
LARCs (continuers and switchers)	2 (2.5)	1 (1.2)	3 (1.8)
Short-acting reversible contraceptive (switchers)	33 (41.3)	28 (32.5)	61 (36.8)
Tubal ligation (switchers)	0 (0)	0 (0)	0 (0)

Abbreviation: LARC, long-acting reversible contraceptive.

### Provider Perspectives

At the 18 health facilities, we conducted 64 routine supportive supervisory visits (33 in Nampula and 31 in Sofala) over the 6-month period, with an average of 3–4 visits per facility. Table 9 shows the number of provider responses to questions in the supportive supervision checklist related to ease and time taken to record data in the standard removal addendum. One trained health care provider who was on-call at each site at the time of the supervision visit responded to the questions in the checklist.

**TABLE 9.** Health Care Provider Reported Ease and Time Taken in Recording in the Removal Addendum at Each LARC Removal Visit, Nampula and Sofala Provinces, Mozambique, December 2018–May 2019

	**Nampula (n=33)** **No. (%)**	**Sofala (n=31)** **No. (%)**	**Total (N=64)** **No. (%)**
Ease in recording			
No problems	27 (81.8)	31 (100)	58 (90.6)
Difficulty	3 (9.1)	0 (0)	3 (4.7)
Did not respond	3 (9.1)	0 (0)	3 (4.7)
Time taken, minutes/visit			
≤2	9 (27.3)	20 (64.5)	29 (45.3)
3–5	17 (51.5)	8 (25.8)	25 (39.1)
6–9	5 (15.2)	0 (0)	5 (7.8)
Did not respond	2 (6.1)	3 (9.7)	5 (7.8)

Abbreviation: LARC, long-acting reversible contraceptive.

#### Recording Removal Data in the FP Addendum

During supervision visits, the majority of providers (91%) reported no challenges in recording the requested information in the standard removal addendum after each client visit. One provider at a Nampula health center explained that the clarity and guidance offered during the training and in the addendum instructions offset any initial recording challenges. Two providers in Nampula and 1 in Sofala explained that recording was not difficult “with the help of the codes.”

*All [columns in the addendum] are easy [to enter required information]*. —Health care provider, Sofala province

Despite this, 1 provider at a Nampula health center noted some initial difficulty in completing the fields for assessing duration of use:

*The date of insertion field [is difficult to record accurately] when women forget to bring the FP card.* —Health care provider, Nampula province

#### Time Taken

Of the 64 provider responses, 29 (45%) reported completing the standard removal addendum within 2 minutes per client visit, while 25 (39%) reported completing it in 3–5 minutes. Only 5 providers (8%) in Nampula reported needing more than 5 minutes. Over time, familiarity with coding contributed to the completion of the forms in fewer minutes:

*In the beginning [recording] was confusing because of the codes, but now it is easy.* —Health care provider, Nampula province

#### Perceived Benefits

During the supervision visits, some providers took the opportunity to state what they perceived to be benefits of the LARC removal indicators, especially capturing data on the number and timing of removals, recognizing reasons for removals, and garnering insights regarding the quality of services. Some responses that providers mentioned about the benefits of collecting this information:

*To know the reasons why women opt to remove the methods before time [method expires].* —Health care provider, Sofala province

*To understand the quality of the services.* —Health care provider, Nampula province

The addendum also reinforced the importance of documenting LARC removals:

*Now we will know exactly how many people removed a LARC method.* —Health care provider, Nampula province

#### Indicators (Inclusion/Exclusion)

Overall, health care providers did not recommend excluding any of the LARC removal indicators; only 1 provider at the Nampula provincial hospital proposed removing the field that records whether the client was counseled on FP after removal, chose a method, and/or was referred, as this was seen as duplicative of the removal outcome field. Furthermore, providers at 3 health facilities suggested additional indicators.

*[We] should have a field to record the method women want removed but after the counseling changed their mind.* —Health care provider, Nampula province

Others suggested indicators related to HIV status and reason for switching methods. It is important to note that HIV status is already included as a field in the FP consultation forms.

## DISCUSSION

### Implications for Quality of Care

We designed this study to test the introduction of LARC removal indicators into Mozambique’s national FP register and assess their feasibility and usefulness for strengthening the delivery of FP services. Findings gleaned from the collection, synthesis, and interpretation of the LARC removal indicators demonstrated several quality-of-care implications. First, a large proportion of clients in this study sought removal services because they reported experiencing negative side effects or bodily changes that they could not control (25.8%), desiring a pregnancy (22.9%), or preferring another contraceptive method (7.9%). Previous studies have pointed to “nuisance” vaginal bleeding as playing a major role in prompting women to seek removals.^9^^,^^10^^,^^17^^,^^18^ In a recent systematic review, Coombe et al.^19^ identified LARCs’ high efficacy and long-term protection as positive qualities enabling women to seek and retain LARCs, although negative impacts on the body persuade some women to seek early removals. They argued that client-provider discussions must include both the positive and negative qualities of LARCs, particularly side effects, to help women make informed method choices and anticipate and adjust to bleeding changes, which can help reduce early removals. For example, the majority (88%) of the 112 removal clients in our study who opted not to go through with their removals following counseling had been using their method for 3 months or less, and 45% reported side effects as the primary reason for seeking removal. This further demonstrates the potential for targeted counseling to address specific client concerns about side effects, supports similar evidence from a Brazilian randomized clinical trial,^11^ and underscores the importance of providing women full information and counseling to enable them to make an informed choice.

The data collected from the FP register addendum also highlight clients’ misconceptions about use-effectiveness periods. Nearly 30% of removal clients claimed “on schedule/expired” as the primary reason for seeking a LARC removal, whereas a much smaller proportion of clients actually had their method removed at the time of method expiration (5.6%) or after the expiration month (7.1%). In addition, 18 (8%) of the women who reported “on schedule/expired” as their reason for seeking removal ultimately chose not to have their method removed after counseling. This finding indicates that during the first client-provider interaction, product duration of effectiveness must be communicated clearly and reiterated in subsequent interactions to dispel misperceptions about the method becoming ineffective before its actual effectiveness end date. These interactions could support retention for those who wish to continue avoiding pregnancy, as noted in other studies.^20^^–^^22^ Providing clear information about duration of effectiveness would also help prevent clients from retaining their method after expiration.

During the first client-provider interaction, product duration of effectiveness must be communicated clearly and reiterated in subsequent interactions to dispel misperceptions about the method becoming ineffective before its actual effectiveness end date.

The synthesis and interpretation of the FP outcome indicator results contributed to a broader understanding of effective FP counseling approaches at the time of removal and areas for improvement. Of the 674 clients whose LARC was successfully removed, 233 (35%) adopted another contraceptive, 39 of whom (16.7%) continued with a LARC method, indicating that for some users LARCs remained their preferred method. Additionally, the fact that 188 clients were not counseled after removal initially raised concerns about quality of care. An examination of clients’ reasons for removals, however, showed that two-thirds of those clients opted for removals because they wanted to get pregnant, easing but not erasing concerns about quality standards within the FP program. Still, it was critical to address quality-of-care issues at the health facilities where clients did not receive any FP counseling. Technical support was provided during targeted supervision visits to reinforce national protocols for FP service provision, which includes systematic FP counseling at the different stages of client interaction.

As evident from the findings, quality counseling at all points of contact, including counseling before insertion of LARCs and at the time of removal, remains important. Studies have demonstrated that clients may switch methods without adequate counseling that addressed all potential side effects.^23^^–^^24^ While dissatisfaction with their LARC did not discourage some women in our study from switching to another method, our results corroborated other studies^9^^,^^23^^,^^24^ in underscoring the imperative of robust counseling on side effects and their management in the initial and subsequent client-provider interactions.

### Tracking Difficult Removals and Referrals

As noted previously, data on LARC removals are not captured in health facility registers nor the national HMIS in Mozambique. Collecting data on LARC removal outcomes inclusive of varying levels of difficulty would provide valuable information for health management teams, particularly when noticing an increase in difficult removals, highlighting the need to investigate technical competencies and the availability of appropriate supplies and medical instruments. In this study, health care providers recorded only 4 difficult removals and 5 removals attempted but not completed. This underscores that difficult removals are rare. However, despite this small number, tracking referrals for difficult removals was a challenge. Although it is important to confirm that LARCs are successfully removed, our study findings were disappointing and highlighted programmatic challenges with tracking referred cases. None of the 6 registered referrals were successfully tracked to the district or provincial hospital due to lack of a medical record number and/or referral form. In addition, referral information was not recorded for the other 3 “attempted but not removed” clients. To improve the delivery of difficult removal services, these registration challenges require efforts to strengthen the national health referral system. At the health facility level, program managers can focus on implementing approaches to ensure difficult removal clients receive high-quality care. Such approaches may include deploying trained providers to perform or supervise difficult removals during scheduled technical support visits, incorporating more on-the-job training during supervision visits, scheduling client follow-up visits when skilled providers are available for procedures, and contacting clients to remind them of removal appointments.

To improve the delivery of difficult removal services, registration challenges require efforts to strengthen the national health referral system.

### Using Data From the FP Register Addendums

After sharing study findings with the MOH, the government has taken initial actions. The MOH, with support from USAID and IFPP, created an informational poster for clients to raise awareness about the use of LARCs, their benefits and possible side effects, the duration of effectiveness for each method, as well as where and when to get LARCs removed. Furthermore, LARC removal is now a dedicated topic addressed in all FP provider trainings. In terms of data use to improve the quality of care, we presented findings to health managers for interpretation and discussion during monthly meetings at the facility level. This systematic synthesis and interpretation of the removal indicators have the potential to contribute to evidence-based guidance regarding monitoring of LARC program achievements, challenges, and training needs. For example, the cases of unsuccessful removals signaled a problem in lack of technical capacity requiring further investigation. Given this experience, we recommend leveraging the regularly scheduled meetings at the facility and district/provincial levels to systematically include a review of the aggregated data from the FP register and addendum so that problems can be identified and appropriate actions can be taken in a timely manner.

### Provider Perspectives

Health care providers interviewed in this study perceived the addendum as easy to record data and useful for understanding how to improve the quality of care. In addition, many providers were able to complete the additional requested information within 2 minutes. Assessing the ease with which health care providers were able to record and understand the removal indicator fields is important to consider during the decision-making process for revising the national FP register. In Mozambique, previous revisions of the FP register book were preceded only by short pilot-testing of the new indicators and tools. This study offers evidence from providers’ experiences of using the addendum for 6 months that supports the feasibility of including the new removal indicators.

### Integrating LARC Removal Indicators

Findings from both the data collected from the addendum and the provider interviews support the integration of LARC removal indicators into the national HMIS to strengthen monitoring of the FP program and the delivery of high-quality FP care. Recognizing the current systemic barriers to tracking difficult removals and referrals as well as the challenges in introducing additional indicators to the HMIS due to space, time, and cost constraints, we recommend 3 new indicators for inclusion: removal outcome; reason for removal; and duration of use (3 months or less; over 3 months). We prioritize these indicators because they highlighted issues of quality of care and the relative ease of collecting and interpreting the data by providers and district health teams. In terms of the FP outcome indicator, because the method uptake portion is already captured in the HMIS, we recommend that FP managers focus on implementation approaches to ensure balanced and complete FP counseling is provided in all client-provider interactions so that clients have the information needed to make free and informed choices.

We prioritize these indicators because of how they highlighted issues of quality of care and the relative ease of collecting and interpreting the data by providers and district health teams.

Supported by study findings, in April 2021, the MOH’s National FP Program initiated revisions to the national FP register book and monthly summary forms, with the support of the FP Technical Working Group. The group, comprised of IFPP, other implementing partners, and donors working on FP programs, as well as monitoring and evaluation experts in other technical areas including maternal and child health, nutrition, and adolescent health, is in the process of weighing the potential integration of the 3 LARC removal indicators recommended by IFPP and the study team. Approval and finalization of the revised FP HMIS are anticipated later in 2022.

### Limitations

This study has methodological limitations. First, data were extracted from FP registers and addendums, therefore data quality and consistency depend on the quality of the recorded data, which can be negatively affected by varying cultures of data management and supervision at each facility. In addition, extracted data were gathered during clinical consultations and directly related to the information the client provided, and therefore could suffer from recall bias. For example, overall, nearly 30% of clients reported “on schedule/expired” as the reason for seeking removals, whereas our analysis documented far fewer clients whose method was removed on schedule or after expiration. This discordance suggests that there may be other reasons for seeking removal that clients do not share. We believe that this type of discordance may be avoided by strengthening provider training in quality counseling, including eliciting and recording reasons for seeking removals.

## CONCLUSION

In summary, the study results illustrate how LARC removals data highlight quality-of-care issues in FP service delivery and provide encouraging insights on the feasibility and utility of including LARC removal indicators in Mozambique’s national HMIS. Findings raised quality-of-care issues that must be addressed at the provider-client nexus and at aggregate levels (clinic, district, province, and national). Study results build on previous reports^9^^,^^23^^,^^24^ to emphasize the importance of routine FP counseling and service provision during all provider-client interactions, including before insertion and at the time of LARC removal. Health care providers must be fully knowledgeable to transmit factually correct information on bleeding changes, side effects, use-effectiveness periods of each method, and the availability of LARC removals. Health information systems must also be able to document all LARC removals, just as they do for insertions. Findings from the study also described the benefits and ease of collecting, synthesizing, and interpreting removals data, except for tracking outcomes of difficult removal referrals. The LARC removal indicators tested in this study are recommended as a means of identifying problems and improving the quality of care for FP clients.

## Supplementary Material

21-00252-Lee-Supplement2.pdf

21-00252-Lee-Supplement3.pdf

21-00252-Lee-Supplement1.pdf

## Translation

**Apresentando indicadores de remoção de contraceptivos de longa duração em um estudo piloto em Moçambique: dinâmica da descontinuação e implicações para a qualidade do atendimento**

**Principais achados**

- A síntese e a interpretação dos dados de remoções de métodos contraceptivos reversíveis de longa duração (MLD) ressaltaram a necessidade de fortalecer o aconselhamento de planeamento familiar (PF), particularmente sobre os efeitos colaterais do método e os períodos de eficácia de uso, em todas as interações cliente-provedor.
- A percepção dos provedores de saúde é de que "a adenda com indicadores de remoção de MLD" é fácil de usar e útil para a compreensão de questões de qualidade de cuidados.

**Principais implicações**

- As partes interessadas nacionais e os implementadores do programa devem garantir aconselhamento de PF de rotina e robusto em todas as interações cliente-provedor, incluindo na pré-inserção de MLD e no momento da remoção, para melhor empoderar as mulheres a fazerem escolhas informadas sobre o método.
- Os governos devem considerar a inclusão de indicadores de remoção de MLD em seus sistemas nacionais de informação de saúde para fortalecer o monitoramento do programa de PF e a prestação de cuidados de PF de alta qualidade.

**RESUMO**

**Introdução:** O recente aumento no uso de contraceptivos reversíveis de longa duração (MLD) resultará em um aumento igual na necessidade de remoções. Poucos países monitoram o número de remoções de MLD e menos ainda rastreiam os motivos para remoção, descontinuação e troca de método. Conduzimos um estudo de método misto para testar a introdução de 5 indicadores de remoção de MLD em Moçambique, avaliar as percepções dos provedores sobre a viabilidade e utilidade do rastreamento desses indicadores e rever os resultados para verificar a qualidade dos cuidados. Os indicadores foram (1) motivo para a remoção, (2) duração do uso, (3) resultado da remoção, (4) motivo do encaminhamento e (5) resultado do planeamento familiar (PF).

**Métodos:** O estudo utilizou uma técnica de amostragem de múltiplos estágios intencional para selecionar 19 unidades sanitárias de intervenção nas províncias de Nampula e Sofala. Durante um período de 6 meses, extraímos dados sobre os 5 indicadores de uma adenda de registro de PF para determinar quando e porquê as utentes buscam remoções de MLD e identificar problemas de qualidade de serviço que precisam ser tratados no programa de PF. Também usamos um checklist de supervisão de apoio para avaliar o tempo gasto para registrar os dados sobre os indicadores e os benefícios e desafios percebidos encontrados pelos provedores durante a manutenção de registros.

**Resultados:** 795 clientes procuraram os serviços para a remoção de MLD; 112 mulheres (14,1%) optaram por não prosseguir com a remoção após aconselhamento direcionado. Os motivos mais relatados para a remoção foram: o método estava dentro do prazo para remoção ou fora do prazo de validade (29,5%), queixas de efeitos colaterais (25,8%) e desejo de engravidar (22,9%). Os provedores de saúde não relataram grandes desafios no registro de informações na adenda. As razões e o momento da remoção apontaram para a necessidade de fortalecer o aconselhamento de PF.

**Conclusão:** Coletar, sintetizar e interpretar os dados dos indicadores de remoção de MLD foi viável e forneceu informações valiosas para aprimorar a qualidade dos cuidados para melhorar a saúde reprodutiva das mulheres e as opções de contraceptivos.

**Peer Reviewed**

**First published online:** January 13, 2022.
