# Supplementary material for: Introducing Long-Acting Contraceptive Removal Indicators in a Pilot Study in Mozambique: Dynamics of Discontinuation and Implications for Quality of Care
Source: Glob Health Sci Pract. 2022 Feb 28;10(1):e2100252. doi: 10.9745/GHSP-D-21-00252 (PMC8885348; doi:10.9745/GHSP-D-21-00252)
Supplement: 21-00252-Lee-Supplement2.pdf [file 21-00252-Lee-Supplement2.pdf]

Supplement to: Jacinto A, Viola Ronda A, Lee C, Fikree FF, Ramirez-Ferrero E. Introducing long-acting contraceptive removal indicators in a pilot study in Mozambique: dynamics of discontinuation and implications for quality of care. Glob Health Sci Pract. 2022;10(1):e2100252. https://doi.org/10.9745/GHSP-D-21-00252

Appendix 2: Supportive Supervision Checklist

My name is______________ and I am a Monitoring and Evaluation Officer with the Integrated Family Planning Project at Pathfinder International. As part of the Implant Removal Study, we are including a short checklist to enable us to understand the ease and/or difficulty you might have faced in completing the FP addendum register for standard removals.

You are requested to take part as the service provider responsible for implant removals in this facility. The checklist will take up to five to ten minutes. There are no right or wrong answers. We will take notes. You are free to choose whether to take part in responding to the checklist. If you choose not to take part, you will not be negatively affected in any way. If you choose to participate, you may stop at any time without any penalty. We will protect all information about you and your participation in responding to this checklist to the best of our ability. We will not take your name. Your name will not appear in any report.

Statement of Consent: I have read/heard the above information and have received answers to any questions I asked. I consent to take part in responding to the checklist.

Your Name (printed) ________________________

Verbal consent taken (for interview): **YES** **NO**

Supplement to: Jacinto A, Viola Ronda A, Lee C, Fikree FF, Ramirez-Ferrero E. Introducing long-acting contraceptive removal indicators in a pilot study in Mozambique: dynamics of discontinuation and implications for quality of care. Glob Health Sci Pract. 2022;10(1):e2100252. https://doi.org/10.9745/GHSP-D-21-00252

SUPPORTIVE SUPERVISION GUIDELINE

1. During the past month, there were ___ removals at your health facility.

[*M&E Officer: Please verify the total number of removals during the past month; disaggregated by implants and IUD by reviewing the FP STANDARD REMOVALS addendum register*]

1 (a). In your own words, describe how you managed the ___ implant removals. Did you refer? If yes, why did you refer.

1(b). In your own words, describe how you managed the ____ IUD removals. Did you refer? If yes, why did you refer.

2. The Family Planning STANDARD REMOVALS register has the following six fields that we feel are important to understand for removal clients:

- i. Date of insertion
- ii. Date of removal
- iii. Reason for current visit
- iv. Reason for removal
- v. Removal outcome
- vi. Reason for referral

[*M&E Officer: Please show the service provider the above six fields in the FP STANDARD REMOVALS register*]

With regards to these six fields, in your opinion, would you or your colleagues:

a. Consider that these six fields are easy to complete?
b. How much extra time was needed to complete these fields?
c. What are the burdens and benefits for you or your colleagues in completing these six fields?
d. Were there any of these six fields that you or your colleagues found difficult to complete? If yes, please describe, in your own words, why they were difficult to complete.
e. Are there any fields that you would suggest changing? If yes, please probe for which one.
f. Are there any fields that you would suggest including? If yes, please probe for additional fields.

Concluding Remarks:

- Those are all the questions we have for today.
- Please let me know if you have any additional information, issues, concerns and questions.
- Thank you again for your participation!
