# Supplementary material for: Introducing Long-Acting Contraceptive Removal Indicators in a Pilot Study in Mozambique: Dynamics of Discontinuation and Implications for Quality of Care
Source: Glob Health Sci Pract. 2022 Feb 28;10(1):e2100252. doi: 10.9745/GHSP-D-21-00252 (PMC8885348; doi:10.9745/GHSP-D-21-00252)
Supplement: 21-00252-Lee-Supplement1.pdf [file 21-00252-Lee-Supplement1.pdf]

Supplement to: Jacinto A, Viola Ronda A, Lee C, Fikree FF, Ramirez-Ferrero E. Introducing long-acting contraceptive removal indicators in a pilot study in Mozambique: dynamics of discontinuation and implications for quality of care. Glob Health Sci Pract. 2022;10(1):e2100252. https://doi.org/10.9745/GHSP-D-21-00252

Family Planning Register Addendum (Standard Removals)

Province:_______________________________ **District:**____________________________ **Name of HF:**________________________

Month:_____________ **Total of SRH consultation :**________________________ **Total number of new PF acceptors:**_______________________________ **Total numbers of PF repeat acceptors:**_____________

Initiators pills:___________ **Initiators of injectable:**______________**Initiators of implantes:**____________**Initiators of IUD:**__________________**Tubal ligation:**______________

#	ID	Date of current visit	Age	Marital status	Parity	Reasons for removal	Date of insertion	Removal outcome	Date of removal	Method removed			Name of referral facility	Reasons for referral	Family planning	FP method uptake	Name of health provider
										Jadele	Implanon	IUD					

CODES:

Marital Status:
1. Married
2. Living in union
3. Divorced/Separated
4. Widowed
5. Not married or living in union

Reasons for Removal:
1. Has expired
9. Husband/in-laws opposition
2. Change
to another method
10. Mother's opposition
3. Opted to get pregnant
11. Infrequent or no sex
4. Vaginal bleeding (spotting, heavy)
12. Reduces sexual pleasure
5. Vaginal discharge (severe)
13. Interferes with body natural processes
6. Arm discomfort/pain
14. IUD/Implant failed (got pregnant)
7. Headache
15. IUD expulsion/partial expulsion
8. Backpain
16. Others (Specify)

Removal outcome:
1. Not removed – counseled
2. Removed – No dificulty
3. Removed – with dificulty
4. Removed – but incompleted (attempted but not removed)
5. Removed – significant difficulty (U/S; surgery)
6. Not removed – referred for removal

Family Planning:
1. Not Removed – counseled
2. Not Removed – referred
3. Not counselled
4. Counseled only
5. Counseled and accepted a method
6. Counseled and referred for a method
7. Counseled and refused a method

Date of insertion:
1. Copy date from client record
2. If client dont have record ask month and year of insertion
3. If client does not remember ask how many months ago
4. Does not know the date of insertion

Reason for referrals:
1. Trained provider unavailable
2. No equipment/consumables available
3. Implant not palpable
4. IUD String not visible
5. Incomplete removal
6. Removal too complicated (additional equipment/expertise required)
7. Client requested referral
8. Others

FP Method Uptake:
1. Female condom
2. Oral contraceptive
3. Injectable
4. Emergency Contraceptive
5. IUD
6. Implant (Jadelle)
7. Implant (Implanon)
8. Tubal ligation
