# Supplementary material for: Introducing Long-Acting Contraceptive Removal Indicators in a Pilot Study in Mozambique: Dynamics of Discontinuation and Implications for Quality of Care
Source: Glob Health Sci Pract. 2022 Feb 28;10(1):e2100252. doi: 10.9745/GHSP-D-21-00252 (PMC8885348; doi:10.9745/GHSP-D-21-00252)

Supplement to: Jacinto A, Viola Ronda A, Lee C, Fikree FF, Ramirez-Ferrero E. Introducing long-acting contraceptive removal indicators in a pilot study in Mozambique: dynamics of discontinuation and implications for quality of care. Glob Health Sci Pract. 2022;10(1):e2100252. https://doi.org/10.9745/GHSP-D-21-00252

Supplement 3. Decision Tree for Completing the FP Outcome Columns in the Standard Implant Removal Addendum

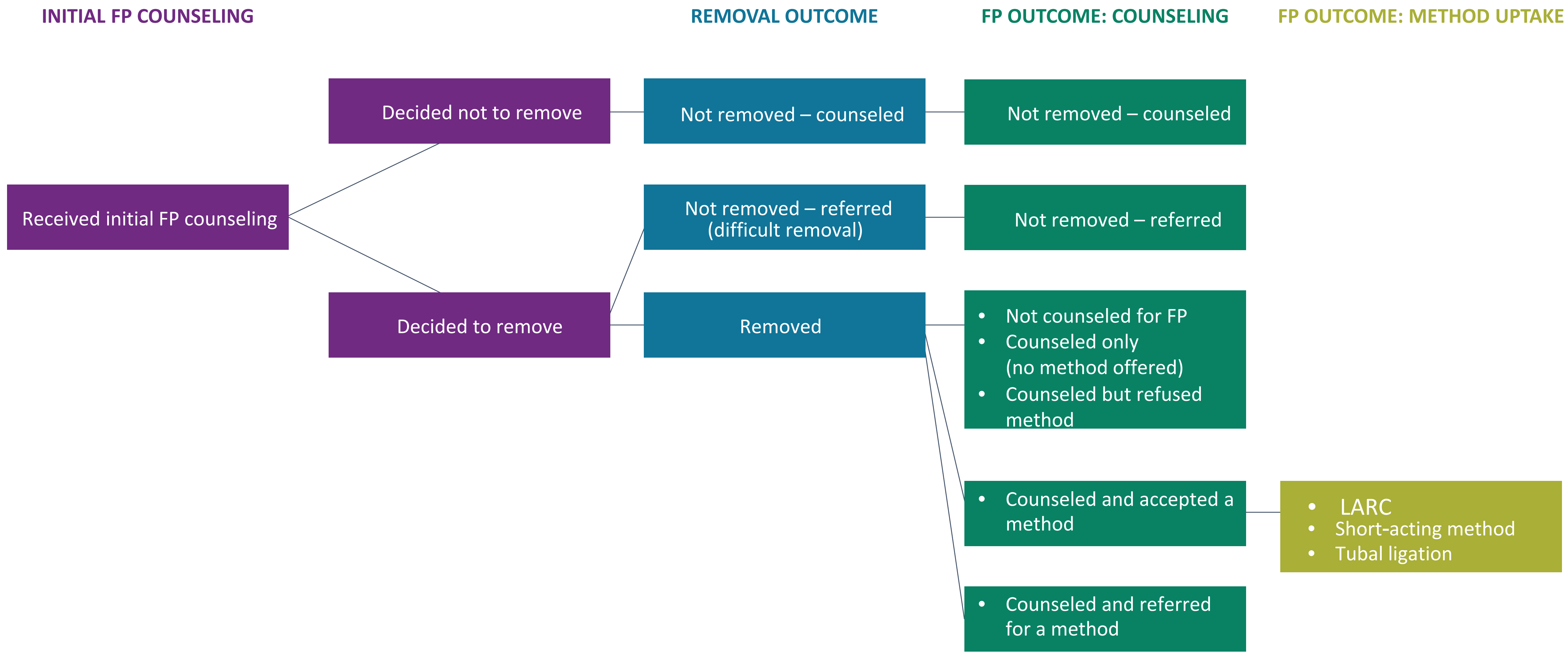

Supplement: 21-00252-Lee-Supplement3.pdf [file 21-00252-Lee-Supplement3.pdf]
